# Granulocyte-macrophage colony-stimulating factor (GM-CSF) shows therapeutic effect on dimethylnitrosamine (DMN)-induced liver fibrosis in rats

**DOI:** 10.1371/journal.pone.0274126

**Published:** 2022-09-02

**Authors:** Mrigendra Bir Karmacharya, Binika Hada, So Ra Park, Kil Hwan Kim, Byung Hyune Choi

**Affiliations:** 1 Department of Physiology and Biophysics, Inha University College of Medicine, Incheon, South Korea; 2 Department of Biomedical Sciences, Inha University College of Medicine, Incheon, South Korea; 3 Veterans Medical Research Institute, Veterans Health Service Medical Center, Seoul, South Korea; Harvard Medical School, UNITED STATES

## Abstract

This study was undertaken to investigate the inhibitory effects of granulocyte-macrophage colony-stimulating factor (GM-CSF) on dimethylnitrosamine (DMN)-induced liver fibrosis in rats. Liver fibrosis was induced in Sprague-Dawley rats by injecting DMN intraperitoneally (at 10 mg/kg of body weight) daily for three consecutive days per week for 4 weeks. To investigate the effect of GM-CSF on disease onset, GM-CSF (50 μg/kg of body weight) was co-treated with DMN for 2 consecutive days per week for 4 weeks (4-week groups). To observe the effect of GM-CSF on the progression of liver fibrosis, GM-CSF was post-treated alone at 5–8 weeks after the 4 weeks of DMN injection (8-week groups). We found that DMN administration for 4 weeks produced molecular and pathological manifestations of liver fibrosis, that is, it increased the expressions of collagen type I, alpha-smooth muscle actin (α-SMA), and transforming growth factor-β1 (TGF-β1), and decreased peroxisome proliferator-activated receptor gamma (PPAR-γ) expression. In addition, elevated serum levels of aspartate aminotransferase (AST), total bilirubin level (TBIL), and decreased albumin level (ALB) were observed. In both the 4-week and 8-week groups, GM-CSF clearly improved the pathological liver conditions in the gross and histological observations, and significantly recovered DMN-induced increases in AST and TBIL and decreases in ALB serum levels to normal. GM-CSF also significantly decreased DMN-induced increases in collagen type I, α-SMA, and TGF-β1 and increased DMN-induced decreases in PPAR-γ expression. In the DMN groups, survival decreased continuously for 8 weeks after DMN treatment for the first 4 weeks. GM-CSF showed a survival benefit when co-treated for the first 4 weeks but a marginal effect when post-treated for 5–8 weeks. In conclusion, co-treatment of GM-CSF showed therapeutic effects on DMN-induced liver fibrosis and survival rates in rats, while post-treatment efficiently blocked liver fibrosis.

## Introduction

Liver fibrosis due to chronic liver injury is a major cause of mortality [1], and a global study reported that liver fibrosis accounted for 2.2% of deaths [2]. Liver fibrosis is a wound-healing response to chronic liver injuries resulting in excessive accumulation of extracellular matrix (ECM) proteins in hepatic tissues. Progressive liver fibrosis advances to cirrhosis, liver failure, portal hypertension, and eventually hepatic dysfunction [3]. Various etiologies such as chronic viral hepatitis, fat accumulation, alcoholic and nonalcoholic hepatic injuries, and toxin/drug-induced metabolic or autoimmune diseases that cause repeated damage to liver tissue have been implicated in liver fibrosis [4, 5]. Dimethylnitrosamine (DMN) is a well-known hepatotoxin that induces liver fibrosis in rats [6], and the DMN-induced liver fibrosis model closely resembles liver damage development in humans, which includes nodule generation, ascites, ECM deposition, biochemical alterations, and histopathological manifestations [7].

Pathophysiologically, chronic hepatic injuries initiate the production of various fibrogenic cytokines in liver tissue, and the continued production of fibrogenic cytokines, such as transforming growth factor-beta (TGF-β), connective tissue growth factor (CTGF), and platelet-derived growth factor (PDGF), causes the transdifferentiation of quiescent nonparenchymal hepatic stellate cells (HSCs) into fibrogenic myofibroblast-like cells [8]. This phenotypic transformation of HSCs into the active myofibroblast-like proliferative state triggers the production of a massive amount of ECM proteins, primarily fibrillar collagens, fibronectin, and alpha smooth muscle actin (α-SMA), and ultimately leads to hepatic fibrosis [9–11]. Activated HSCs are also responsible for the proliferation and migration of phenotypically transformed fibroblasts and the binding of TGF-β1 to its receptor, which triggers the migration of such fibroblasts [12]. Binding of TGF-β1 with type II receptor results in the recruitment and phosphorylation of type I receptor and phosphorylates suppressor of mothers against decapentaplegic (Smad) 2 or 3 proteins. Phosphorylated Smad2 and Smad3 bind to Smad4 to form a heterotrimeric complex, which translocates to the nucleus and transcribes genes involved in ECM synthesis and deposition [13]. Due to the importance of TGF-β signaling, ECM synthesis, and HSC transformation in the pathophysiology of liver fibrosis, state-of-the-art antifibrotic strategies that target the ECM [14] and HSCs [15] and stem cell-based therapies [16] that target TGF-β1 [17] and enhance antifibrotic efficacy [18] are currently used to treat liver fibrosis.

Granulocyte-macrophage colony-stimulating factor (GM-CSF) is a multipotent cytokine synthesized by macrophages, lymphocytes, fibroblasts, endothelial cells, and others [19]. GM-CSF has been implicated in a multitude of biological functions such as the chemotaxis of inflammatory cells to wound sites [20], the proliferation and differentiation of early hematopoietic progenitor cells [21], epithelial regeneration [21], and wound-healing and neovascularization [22]. GM-CSF plays a complex tissue-dependent role in fibrosis [23], and has antiviral and immunoregulatory effects in chronic hepatitis B [24]. Furthermore, GM-CSF has been shown to promote hepatic regeneration after 70% hepatectomy by enhancing hepatocellular DNA synthesis in a rat model [25].

Previously, we demonstrated that GM-CSF inhibits glial formation and has a long-term protective effect after spinal cord injury [26]. Additionally, we have shown that GM-CSF has therapeutic potential for the remodeling of vocal fold (VF) wounds and promotion of VF regeneration [20], and the stimulation and mobilization of bone marrow mesenchymal stem cells (MSCs) [27]. Furthermore, in a previous study, we showed that GM-CSF inhibited the TGF-β-induced Rho-ROCK pathway and reduced excessive expression of chondroitin sulfate proteoglycan (CSPG) core proteins in rat primary astrocytes [28]. In the present study, we produced a rat DMN-induced liver fibrosis model and examined the anti-hepatofibrotic effects of GM-CSF on liver fibrosis. The potential mechanisms responsible for the attenuation of liver fibrosis by GM-CSF are discussed.

## Materials and methods

### Chemicals and antibodies

DMN was purchased from Wako Pure Chemical Industries (147–03781, Richmond, VA, USA). Recombinant mouse GM-CSF was obtained from Chemicon (Temecula, CA, USA). Anti-collagen type I antibody (ab34710, rabbit polyclonal), anti-α-SMA antibody (ab5694, rabbit polyclonal), and goat anti-rabbit IgG H&L (HRP) antibody (ab205718) were purchased from Abcam (Cambridge, MA, USA). Anti-β-actin antibody (sc-47778) was purchased from Santa Cruz Biotechnology (Santa Cruz, CA, USA); anti-TGF-β1 antibody was purchased from Sigma (St. Louis, MO, USA); and anti-peroxisome proliferator-activated receptor-gamma (PPAR-γ) antibody (A3409A) was purchased from Thermo Fisher Scientific (Waltham, MA, USA).

### Experimental design

The study was carried out in compliance with ARRIVE guidelines (https://arriveguidelines.org). The experimental protocols for the animal study were approved by the Inha University Institutional Animal Care and Use Committee (INHA-IACUC, approval ID: INHA 170228-484-2). All methods were carried out in accord with relevant guidelines and regulations. All animals were treated strictly following approved protocols. Sixty male Sprague-Dawley rats (8 weeks, 300 g) were purchased from Orient-Bio (Gyeonggi-do, South Korea). Rats were housed in a pathogen-free animal facility under 12 h light/dark cycle at constant temperature and humidity throughout the experiment. All rats were fed with standard rat chow with access to tap water *ad libitum*. After 1 week of acclimatization, animals were assigned randomly into 6 groups (*n* = 10 per group). Liver fibrosis was induced in the rats by intraperitoneal (IP) injection of DMN (10 mg/kg body weight daily) three times weekly for 4 weeks and sacrificed 4 or 8 weeks after commencing DMN administration using carbon dioxide (CO_2_).

For GM-CSF treatment, rats in the 4-week groups received GM-CSF (50 μg/kg body weight daily, IP) from the day of commencing DMN administration twice per week for 4 weeks, whereas rats in the 8-week groups received GM-CSF using the same protocol during the 5^th^ to 8^th^ weeks after commencing DMN administration. Sham controls were administered equal volumes of saline (0.9%, IP). The experimental groups were as follows:

1. Control-4w (saline for 1–4 weeks; sacrificed at the end of the 4^th^ week)
2. DMN-4w (DMN only for 1–4 weeks; sacrificed at the end of the 4^th^ week)
3. DMN+GM-4w (DMN and GM-CSF for 1–4 weeks; sacrificed at the end of the 4^th^ week)
4. Control-8w (saline for 1–4 weeks; sacrificed at the end of the 8^th^ week)
5. DMN-8w (DMN only for 1–4 weeks; sacrificed at the end of the 8^th^ week)
6. DMN+GM-8w (DMN for 1–4 weeks; GM-CSF for 5–8 weeks; sacrificed at the end of the 8^th^ week).

Body weights were measured 3 times per week and once immediately before excising livers.

### Serum biochemical analysis

Blood samples were collected from heart chambers 4 or 8 weeks after commencing DMN administration. Samples were left at room temperature (RT) for 30 min, centrifuged at 3000 rpm for 10 min, and serum was stored at −70°C until required for the analyses of aspartate aminotransferase (AST), albumin (ALB), and total bilirubin (TBIL), which was performed by spectrometry using a Beckman Coulter AU680 Chemistry Analyzer (Beckman Coulter Life Sciences, Ariake Koto-Ku, Tokyo, Japan).

### Histopathological examination

After sacrifice, whole livers were excised, weighed, and fixed in 10% neutral formalin solution. Histopathological slides of tissue samples were prepared by a certified histopathologist. Briefly, fixed liver samples were embedded in paraffin blocks, and 5 μm thick sections were prepared. Paraffin-embedded sections were deparaffinized and processed for Sirius Red and hematoxylin-eosin (H&E) staining, which have been previously used to evaluate the progression of liver fibrosis [29, 30].

### Immunohistochemical examination

Thin liver tissue sections with 5 μm thickness were prepared and mounted on slides, deparaffinized in xylene, and rehydrated using an alcohol series. The levels of collagen type I, α-SMA, TGF-β1, and PPAR-γ were determined by immunohistochemical staining using appropriate primary antibodies as described in ’Chemicals and Antibodies’ according to the manufacturer’s instructions.

### Western blot analysis

Total proteins in liver tissues were obtained using RIPA buffer (Thermo Fisher Scientific), according to the manufacturer’s instructions. Liver tissues were homogenized in 300 μL RIPA buffer [0.5% Nonidet P-40, 20 mM Tris-Cl (pH 8.0), 50 mM NaCl, 50 mM NaF, 100 μM Na_3_VO_4_, 1 mM dithiothreitol, 50 μg/mL phenylmethylsulfonyl fluoride] containing protease inhibitors, and homogenates were centrifuged at 13,200 rpm for 30 min at 4°C. Proteins (30 μg) were separated by sodium dodecyl sulfate-polyacrylamide gel electrophoresis (SDS-PAGE) and electrotransferred to polyvinylidene difluoride (PVDF) membranes, which were blocked with non-fat milk solution in Tris-buffered saline containing 0.1% Tween-20 (TBST) for 1 h at RT. Membranes were then incubated with primary antibodies (α-SMA, collagen type I, TGF-β1, or β-actin) at manufacturers’ recommended concentrations overnight at 4°C. After washing, membranes were incubated with horseradish peroxidase (HRP)-conjugated anti-rabbit secondary antibodies directed against primary antibodies for 1 h at RT. Blots were detected using an enhanced Bio-Rad Western blot detection system (Bio-Rad Laboratories, Hercules, CA, USA). The antibodies used were the same as those used for immunohistochemistry.

### Image analysis

Prepared histopathological or immunohistologic slides were examined under a microscope (DMi8, Leica Microsystems Inc., Buffalo Grove, IL, USA), and acquired images were analyzed quantitatively using Image J software [31]. Briefly, acquired images (n = 5 for each group) were deconvoluted, threshold adjusted, and percentage expressions were calculated. Results are presented as means ± standard errors of means.

### Statistical analysis

Statistical analyses were performed using SPSS software (Version 20, IBM SPSS Statistics, IBM Corp., Armonk, NY, USA). Results are presented as means ± SEMs (standard error of means). One-way analyses of variance (ANOVA) followed by Tukey’s *post hoc* test was used to determine the significances of intergroup differences. Statistical significances are represented as ***p ≤ 0.001, **p ≤ 0.01, or *p ≤ 0.05, and ns (not significant).

## Results

### GM-CSF reduced DMN-induced hepatofibrosis

**Fig 1:**
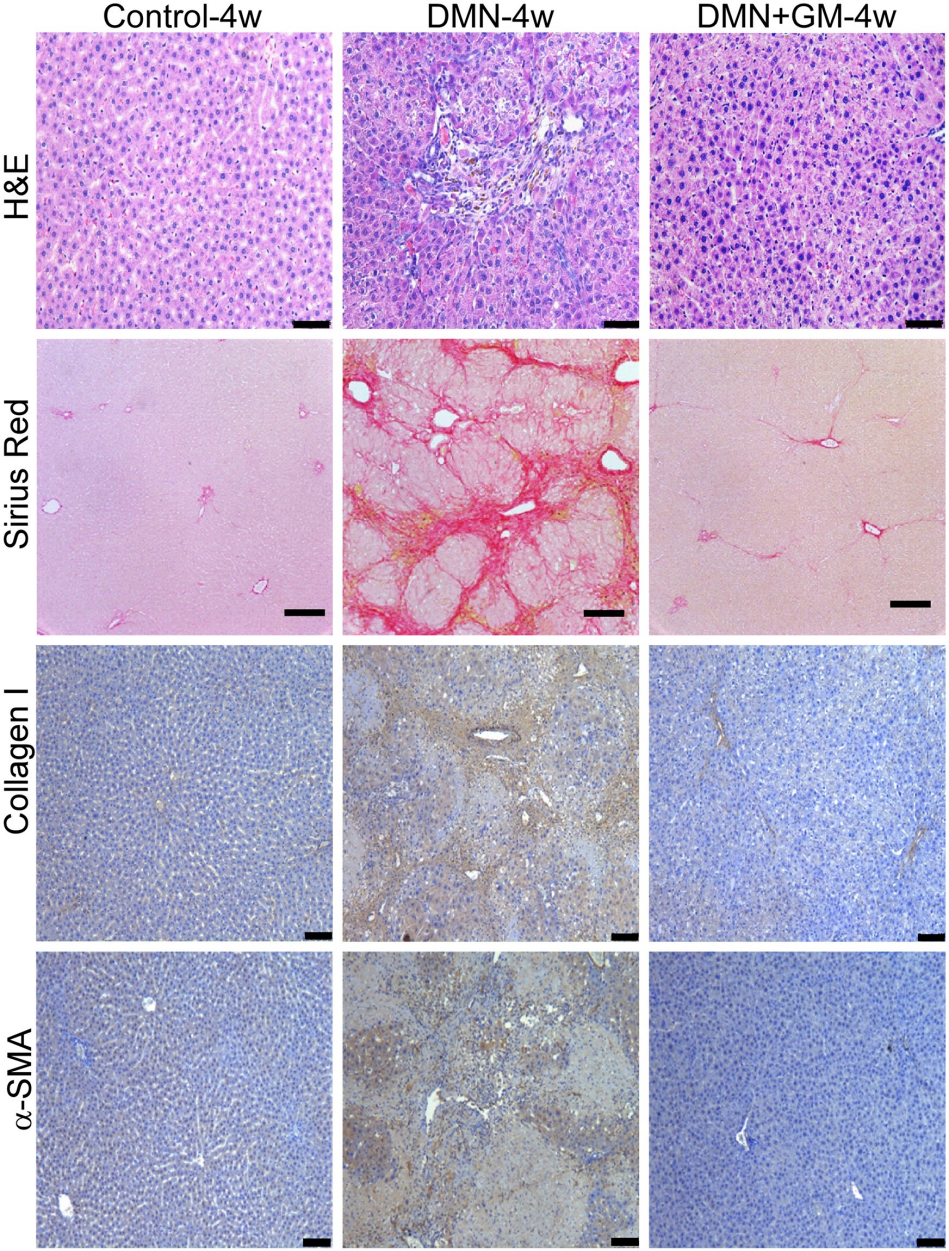
Effects of GM-CSF on DMN-induced histopathological changes in the 4-week group. Liver tissues were collected 29 days after initial DMN administration and fixed in 10% neutral formalin. Thin sections (5 μm) were cut and stained with hematoxylin and eosin (H&E) and Sirius Red. Collagen type I and α-SMA proteins were detected immunohistochemically. Scale bar = 100 μm.

**Fig 2:**
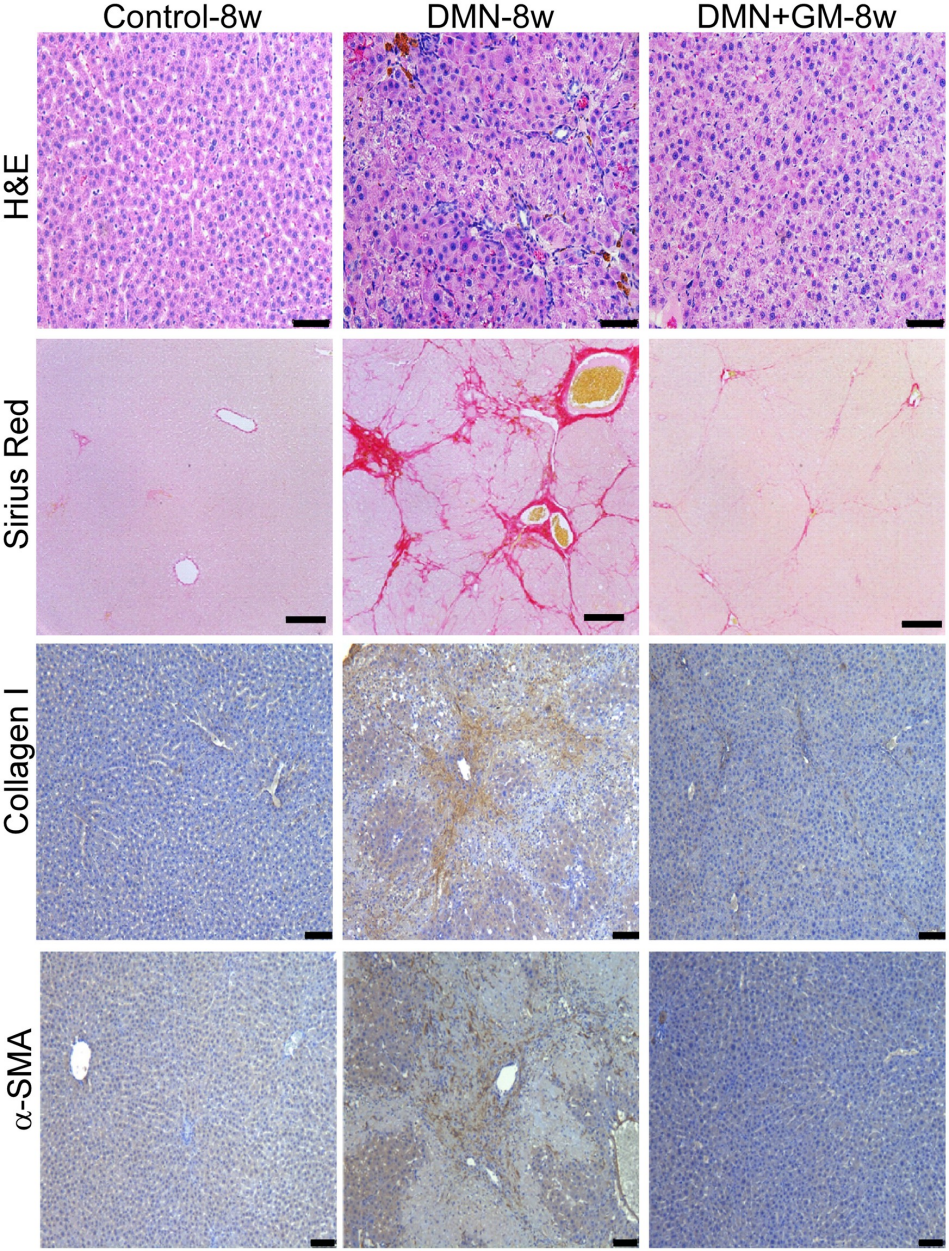
Effects of GM-CSF on DMN-induced histopathological changes in DMN+GM-8w group. Liver tissues were collected 57 days after initial DMN administration and fixed in 10% neutral formalin solution. Thin sections (5 μm) were cut and stained with hematoxylin and eosin (H&E) and Sirius Red. Collagen I and α-SMA proteins were detected immunohistochemically. Scale bar = 100 μm.

DMN administration induced significant hepatofibrotic changes in liver tissues in the DMN-4w and -8w groups. Qualitative examination of H&E-stained slides showed that DMN induced distinct changes in the cellular architecture of liver tissue, which exhibited bands typical of fibrotic liver (Figs 1 and 2). These fibrotic bands were not observed in the corresponding sham-treated groups. In addition, liver sections in the DMN-4w and -8w groups exhibited abnormal hepatic plate arrangement, inflammatory cell infiltration, collagen fiber deposition, and fibrosis (Figs 1 and 2). Sham controls had a normal lobular architecture with structurally intact hepatic lobules and an orderly arrangement of hepatic plates (Figs 1 and 2). DMN-induced hepatofibrosis was further confirmed by elevated collagen expression as determined by Sirius Red staining. Sirius Red-staining showed that DMN increased extracellular collagen deposition in liver tissue in both the DMN-4w and -8w groups (Figs 1 and 2). Furthermore, immunohistochemical staining showed a marked increase in the levels of collagen type I and α-SMA expression in the DMN-treated groups. Both DMN-4w- and -8w groups demonstrated significantly higher collagen type I expression and α-SMA-positive cell numbers than the corresponding sham-treated control groups (Figs 1 and 2).

In the DMN+GM-4w and -8w groups, GM-CSF treatment prevented DMN-induced liver damage. H&E staining showed a normal arrangement of hepatic plates, a reduction in inflammatory cell infiltration, and thickening of collagen bundles in the DMN+GM groups as compared with the corresponding DMN groups (Figs 1 and 2). Sirius Red staining confirmed GM-CSF treatment reduced DMN-induced collagen deposition. Consistent with Sirius staining results, GM-CSF treatment significantly suppressed the DMN-induced expressions of collagen type I and α-SMA in the DMN+GM-4w and -8w groups (Figs 1 and 2).

**Fig 3:**
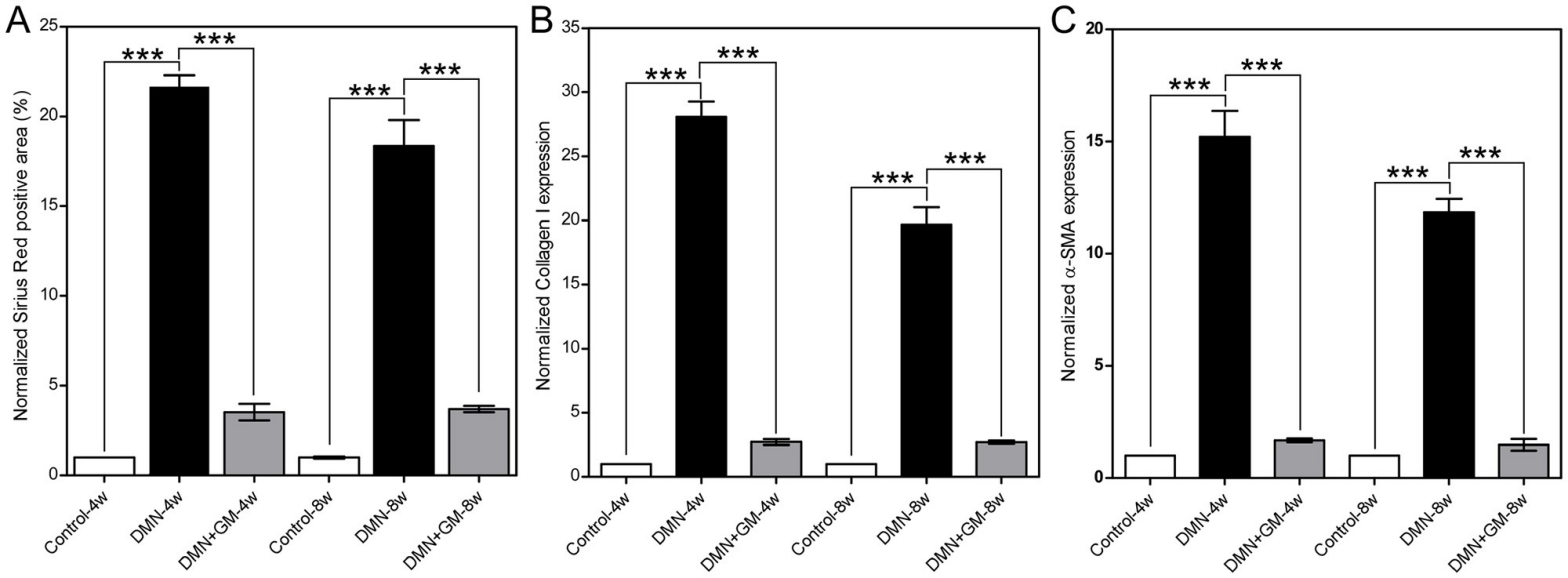
The effects of GM-CSF on DMN-induced histopathological changes. (**A**) Sirius Red staining, (**B**) collagen type I content, and (**C**) α-SMA expression. Data were calculated from liver tissue sections (Figs 2 and **3**). Colors corresponding to Sirius Red, collagen type I, and α-SMA for each rat were separated by deconvolution, and color intensities corresponding to Sirius Red, collagen type I, and α-SMA were measured. Results were averaged and normalized with respect to the corresponding control groups and are presented as fold-changes, expressed as means (± SEM) was significantly different between the control and DMN-treated groups and between the DMN-treated groups and DMN+GM-CSF groups (***p ≤ 0.001).

Sirius-Red-positive cell changes in the DMN-4w and DMN+GM-CSF-4w groups were 22.58 (± 0.59) and 3.52 (± 0.46), respectively, and in the DMN-8w and DMN+GM-CSF-8w groups were 18.36 (± 1.44) and 3.69 (± 0.18), respectively, as compared with corresponding sham-treated control groups (Fig 3A). DMN treatment significantly increased Sirius-Red-positive cell counts in the DMN+GM-4w and -8w groups (*p* ≤ 0.001), and Sirius-Red-positive cell counts were significantly lower in the DMN-4w and -8w groups than in the corresponding DMN+GM-4w and -8w groups (*p* ≤ 0.001).

Likewise, fold increases in collagen type I in the DMN-4w and DMN+GM-4w groups were 28.10 (± 1.16) and 2.71 (± 1.16), respectively, and in the DMN-8w and DMN+GM-8w groups were 19.68 (± 1.34) and 2.69 (± 1.34), respectively, versus corresponding sham control (Fig 3B). Expressions of α-SMA were also 15.21 (± 1.15) and 11.84 (± 0.59) fold higher in the DMN-4w and -8w groups, respectively, than in the corresponding control groups, and these expressions were substantially lower in the DMN+GM-4w and -8w 1.67 (± 0.09) and 1.48 (± 0.26) folds, respectively (Fig 3C). Furthermore, these increases in collagen type I and α-SMA expression levels in the DMN-4w and -8w groups were significant (*p* ≤ 0.001), as were observed reductions in the expressions of collagen type I and α-SMA (*p* ≤ 0.001).

### GM-CSF reduced DMN-induced liver damage and inhibited hepatotoxicity

**Fig 4:**
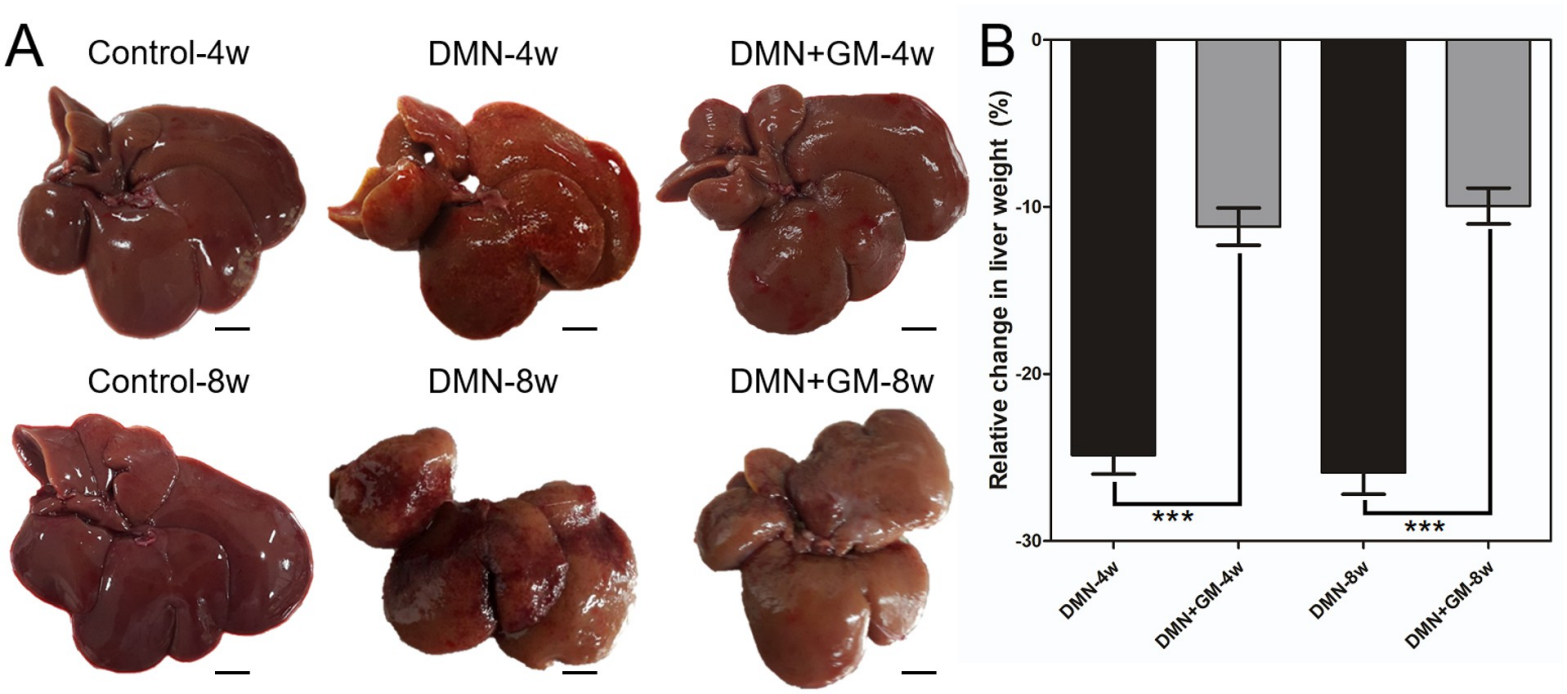
Effects of GM-CSF on DMN-induced changes in liver morphology, texture, and weight. (**A**) Images showing the morphology and texture of liver lobes in the control group, DMN-4w and -8w groups, and DMN+GM-4w and -8w groups. Note that DMN-treatment markedly changed both liver morphology and texture, and that GM-CSF treatment prevented DMN-induced changes in liver morphology and texture in the DMN+GM-4w and -8w groups. Scale bar = 1 cm. (**B**) Histograms showing changes in liver weight with respect to the corresponding controls in the DMN-4w and -8w and DMN+GM-4w and -8w groups. GM-CSF treatment significantly suppressed DMN-induced reductions in liver weight in both the 4- and 8-week treatment groups (***p ≤ 0.001).

Qualitative visual inspection of excised livers showed that the liver lobes in the sham-treated control groups were brown, smooth, and soft with glossy surfaces (Fig 4A). Surfaces of DMN-treated livers were rough, coarse, hard, shrunken, scarred, and dark, whereas livers in the GM-CSF groups had smoother surfaces, an enhanced brown texture, no scars, and a texture similar to that of sham controls. The DMN-4w and -8w groups had significantly lower liver weights, when compared to the decrease of corresponding body weights. Mean liver weight was 3.91 (± 0.09) g in the Control-4w group and 2.94 (± 0.15) g in the DMN-4w group and 3.87 (± 0.06) g in the Control-8w group and 2.66 (± 0.23) g in the DMN-8w group. Percentage decreases in liver weights in the DMN-4w and DMN-8w groups were 24.85 (± 1.13) % and 25.91 (± 1.28) %, respectively, as compared with corresponding sham-treated controls (Fig 4B). GM-CSF significantly reduced DMN-induced liver weight loss. Mean liver weights were 3.58 (± 0.1) g in the DMN+GM-4w group and 3.52 (± 0.12) g in the DMN+GM-8w group. Percentage decreases in liver weights in the DMN+GM-4w and DMN+GM-8w groups were only 11.18 (± 1.12) % and 9.94 (± 1.08) %, respectively, versus corresponding sham controls. The reduction in liver weight loss by GM-CSF in the DMN+GM-4w and -8w groups was significant versus the DMN groups. These results demonstrate that GM-CSF reduced DMN-induced liver damage and inhibited hepatotoxicity.

### GM-CSF improved DMN-induced liver dysfunction

**Fig 5:**
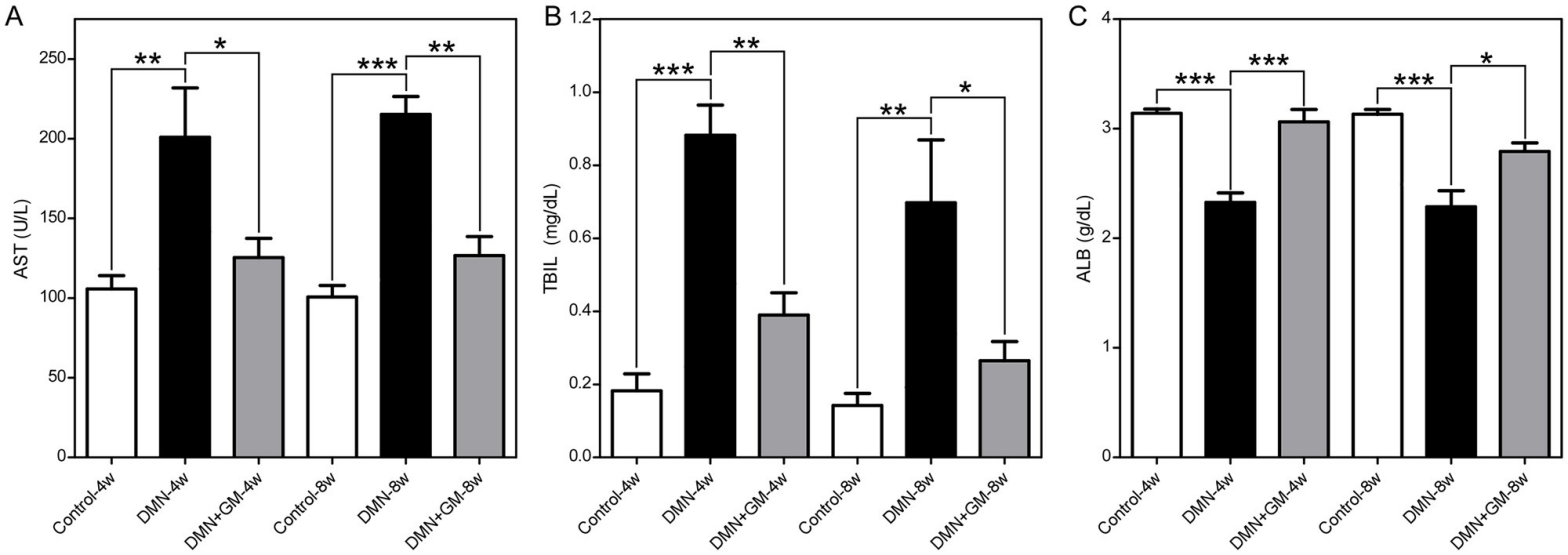
Effects of GM-CSF on DMN-induced hepatotoxicity. Blood samples were collected from venous and arterial blood vessels and heart chambers at 4 or 8 weeks after commencing DMN administration. Aspartate aminotransferase (AST), albumin (ALB), and total bilirubin (TBIL) in blood serum were determined spectrophotometrically. Changes in serum AST (U/L) (**A**), TBIL (mg/dL) (**B**), and ALB (g/dL) (**C**) in the DMN-4w and -8w and DMN+GM-4w and -8w groups. Results are expressed as means ± SEMs. The significances of differences between the control and DMN-treated groups and between DMN-treated groups and DMN+GM-CSF groups are expressed as ***p ≤ 0.001, **p ≤ 0.01, and *p ≤ 0.05.

Biochemical analyses of liver functions showed that rats in the DMN-4w and -8w groups developed hepatic injuries, as evidenced by significantly higher concentrations of AST and TBIL and a significantly lower concentration of ALB compared to sham-treated control rats (Fig 5). GM-CSF treatment prevented DMN-induced liver dysfunction in the DMN+GM-4w and -8w groups. Quantitatively, the mean (± SEM) concentrations of AST (U/L) in the Control-4w, DMN-4w, and DMN+GM-4w groups were 105.8 (± 8.36), 200.9 (± 30.97), and 125.4 (± 12.07), respectively, and in the Control-8w, DMN-8w, and DMN+GM-8w groups were 100.6 (± 7.25), 215.2 (± 11.20), and 126.7 (± 11.88), respectively (Fig 5A). Furthermore, mean (± SEM) concentrations of AST (U/L) in the Control-4w vs. DMN-4w (*p* ≤ 0.01), DMN-4w vs. DMN+GM-4w (*p* ≤ 0.05), Control-8w vs. DMN-8w (*p* ≤ 0.001), and DMN-8w vs. DMN+GM-8w (*p* ≤ 0.01) groups were statistically significant, while those in the Control-4w and DMN+GM-4w groups and the Control-8w and DMN+GM-8w groups were non-significant.

Similarly, the means (±SEMs) of TBIL (mg/dL) for the Control-4w, DMN-4w, and DMN+GM-4w groups were 0.18 (± 0.05), 0.88 (± 0.08), and 0.39 (± 0.06), respectively, and those of the Control-8w, DMN-8w, and DMN+GM-8w groups were 0.14 (± 0.03), 0.69 (± 0.17), and 0.27 (± 0.05), respectively (Fig 5B). Differences between mean (± SEM) TBIL (mg/dL) values of the Control-4w and DMN-4w (*p* ≤ 0.001), DMN-4w and DMN+GM-4w (*p* ≤ 0.01), Control-8w and DMN-8w (*p* ≤ 0.01), and DMN-8w and DMN+GM-8w (*p* ≤ 0.05) groups were all significant, while those between the Control-4w and DMN+GM-4w groups and between the Control-8w and DMN+GM-8w groups were non-significant.

The mean (± SEM) concentrations of ALB (g/dL) in the Control-4w, DMN-4w, and DMN+GM-4w groups were 3.14 (± 0.04), 2.33 (± 0.09), and 3.06 (± 0.11), respectively, and those for the Control-8w, DMN-8w, and DMN+GM-8w groups were 3.13 (± 0.04), 2.29 (± 0.15), and 2.79 (± 0.08), respectively (Fig 5C). Difference between mean (± SEM) concentrations of TBIL (mg/dL) in the Control-4w and DMN-4w (*p* ≤ 0.001), DMN-4w and DMN+GM-4w (*p* ≤ 0.001), Control-8w and DMN-8w (*p* ≤ 0.001), and DMN-8w and DMN+GM-8w (*p* ≤ 0.05) groups were significant, while those between the Control-4w and DMN+GM-4w and Control-8w and DMN+GM-8w groups were non-significant. These results further confirm the hepatoprotective effects of GM-CSF on DMN-induced liver dysfunction.

### GM-CSF improved survival rates and increased the body weights of DMN-treated rats

**Fig 6:**
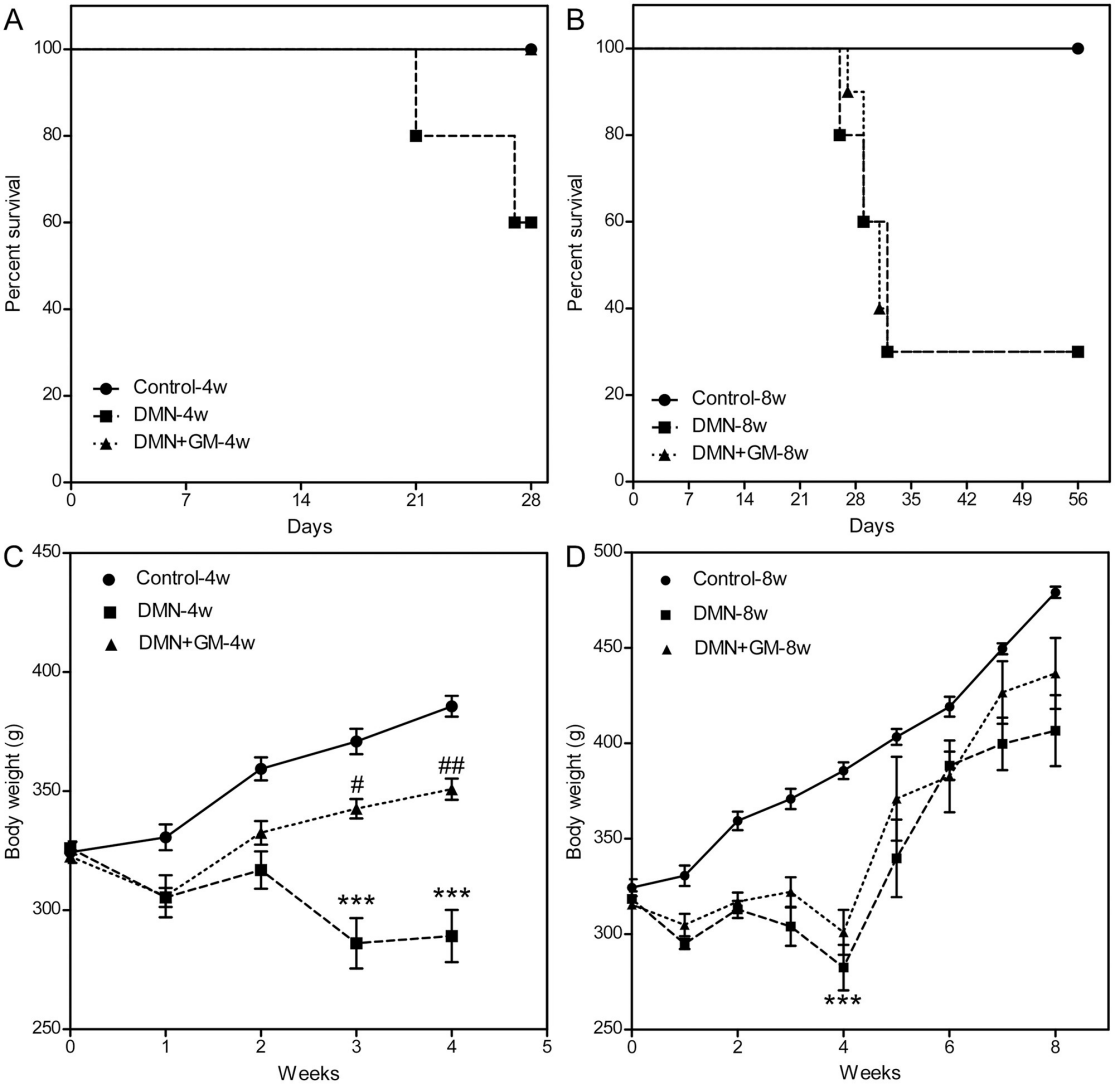
Effects of GM-CSF on DMN-induced changes in survival and body weights. Survivals of sham controls (n = 10), DMN-4w and -8w groups (n = 10), and DMN+GM-4w and -8w groups (n = 10) for (**A**) 4-week groups and (**B**) 8-week groups. Deaths were recorded weekly and used to calculate survival rates. Bodyweights are shown for sham controls, DMN-treated rats, and DMN+GM rats for (**C**) 4-week groups and (**D**) 8-week groups. Bodyweights were measured weekly throughout the study. Results are expressed as means (± SEMs). The significances of differences between the control and DMN-treated groups with or without GM-CSF are expressed as ##*p* ≤ 0.01 and #*p* ≤ 0.05. Significant differences were observed between DMN-treated groups and DMN+GM-CSF groups (*** *p* ≤ 0.001).

Rats in the Control-4w and Control-8w groups survived the experimental period (Fig 6A and 6B). However, 40% of rats in the DMN-4w group and 70% in the DMN-8w group died. Interestingly, GM-CSF significantly increased the survival of rats in the DMN+GM-4w (co-treatment) group but not in the -8w (post-treatment) group. Rats in the DMN+GM-4w group achieved 100% survival (Fig 6A), whereas rats in the DMN+GM-8w group showed no significant difference in the survival rates from the DMN-treated rats (Fig 6B).

Four weeks of DMN administration significantly reduced bodyweights. Rats in the DMN-4w group lost 11.38 (± 4.68) % of bodyweight versus baseline, while bodyweights in the Control-4w group increased by 19.46 (± 2.46) % (Fig 6C). However, GM-CSF treatment prevented DMN-induced bodyweight loss. The DMN+GM-4w group showed an 8.78 (± 1.46) % increase in bodyweight over four weeks, and mean bodyweight in the DMN+GM-4w group (350.8 (± 5.19) g) was significantly higher than in the DMN-4w group (289.17 (± 9.51) g), though baseline bodyweights were comparable (322.5 (± 2.8) g and 326 (± 2.74) g respectively) (Fig 6C). Similarly, mean bodyweight in the DMN-8w group was 11.33 (± 7.12) % lower than at baseline. GM-CSF significantly prevented DMN-induced bodyweight lost in the DMN+GM-4w groups but not in DMN+GM-8w groups (Fig 6D).

### GM-CSF inhibited DMN-induced increases in TGF-β1 protein levels

**Fig 7:**
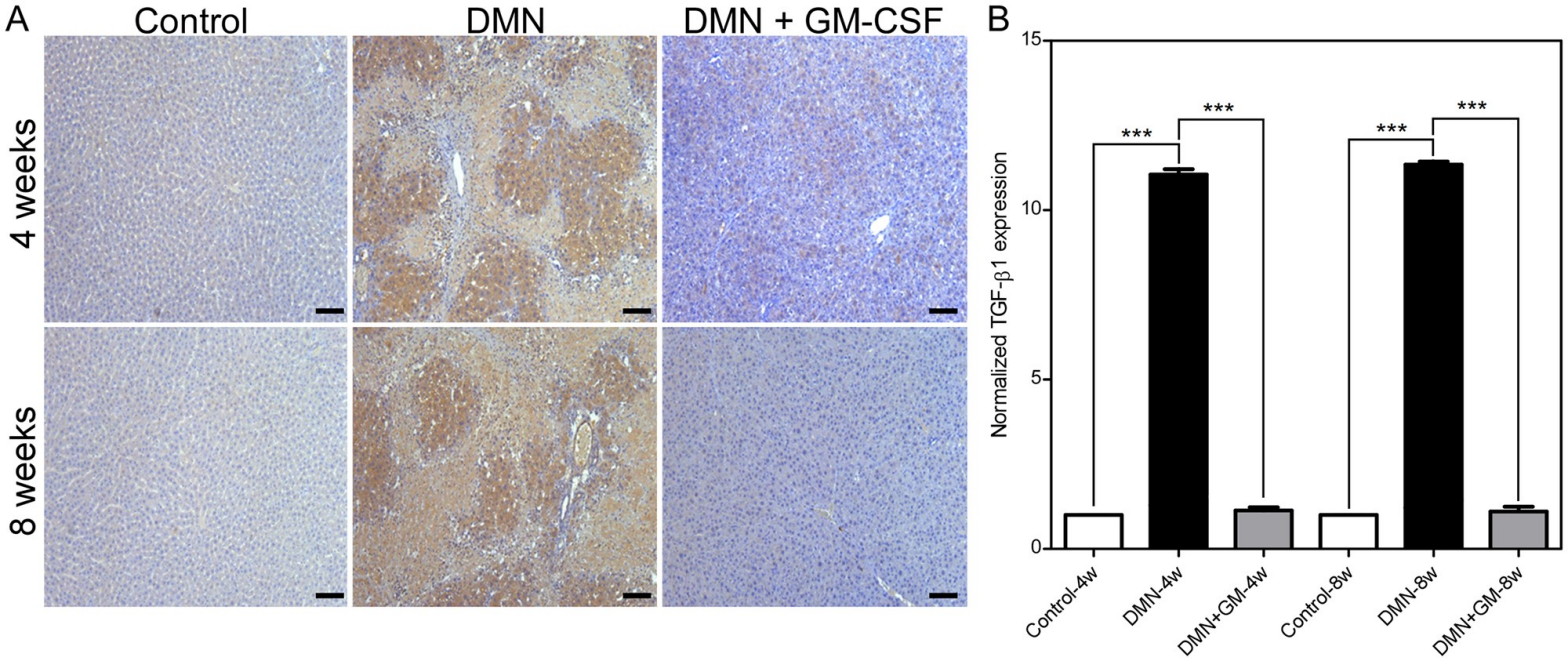
Effects of GM-CSF on DMN-induced TGF-β1 expression. (**A**) Representative images of TGF-β1 immunohistochemical staining in the DMN, DMN+GM, and control 4 and 8-week groups are shown. (**B**) Quantitative analysis of TGF-β1 expressions. Results are expressed as normalized means (± SEM) with respect to controls. Significant differences were observed between the controls and DMN-treated groups and between the DMN-treated groups and DMN+GM-CSF groups (***p ≤ 0.001). Scale bar = 100 μm.

Visual assessments of immunohistochemical slides showed that DMN significantly increased TGF-β1 expression, and that GM-CSF substantially reduced this increase in the DMN+GM-4w and -8w groups (Fig 7A). DMN increased TGF-β1 levels by 11.05 (± 0.16) and 11.34 (± 0.09) fold in the DMN-4w and -8w groups, respectively. However, TGF-β1 levels in DMN+GM-4w and -8w groups were comparable to those in the corresponding control groups (Fig 7B). Differences between TGF-β1 levels in the Control-4w and DMN-4w groups and DMN-4w and DMN+GM-4w groups were significant (*p* ≤ 0.001), but the difference between TGF-β1 levels in the Control-4w and DMN+GM-4w groups was not. Similarly, differences between TGF-β1 levels in the Control-8w and DMN-8w groups and DMN-8w and DMN+GM-8w groups were also significant (*p* ≤ 0.001), but differences between TGF-β1 levels in the Control-8w and DMN+GM-8w groups was not. The lower TGF-β1 levels in GM-CSF-treated groups as compared with corresponding DMN-treated groups suggested GM-CSF has an inhibitory effect on the TGF-β1 pathway.

### GM-CSF suppressed DMN-induced reductions in PPARγ proteins levels

**Fig 8:**
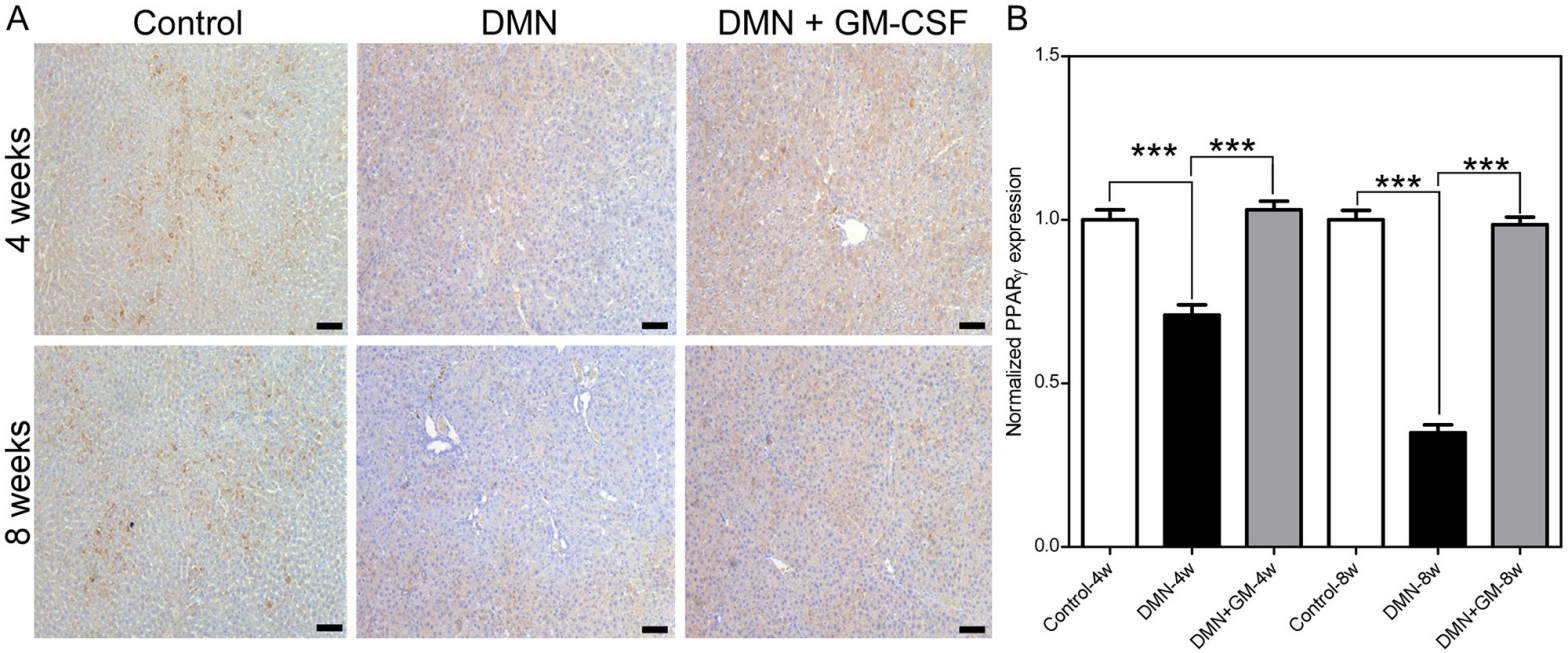
Effects of GM-CSF on the DMN-induced inhibition of PPAR-γ expression. (**A**) Representative images of PPAR-γ immunohistochemical staining in DMN, DMN+GM, and control 4- and 8-week groups are shown. (**B**) Quantitative analysis of PPAR-γ expressions. Results are expressed as normalized means (± SEM) with respect to controls. Significant differences were observed between the control and DMN groups and between the DMN and DMN+GM-CSF groups (***p ≤ 0.001). Scale bar = 100 μm.

DMN significantly lowered PPARγ levels in liver tissues, and GM-CSF co-treatment markedly suppressed this reduction in the DMN+GM-4w and -8w groups (Fig 8A). DMN administration reduced PPARγ levels by 0.71 (± 0.03) and 0.35 (± 0.02) folds in the DMN-4w and -8w groups, respectively, but PPARγ levels in the DMN+GM-4w and -8w groups were comparable to those in the corresponding control groups (Fig 8B). Differences between PPARγ expression levels in the Control-4w and DMN-4w groups and DMN-4w and DMN+GM-4w groups were significant (*p* ≤ 0.001), but the difference between PPARγ levels in the Control-4w and DMN+GM-4w was not. Similarly, differences between PPARγ levels in the Control-8w and DMN-8w groups and DMN-8w and DMN+GM-8w groups were significant (*p* ≤ 0.001), but the difference between PPARγ expression levels in the Control-8w and DMN+GM-8w groups was not.

### Western blot analysis confirmed the effects of GM-CSF on protein levels

**Fig 9:**
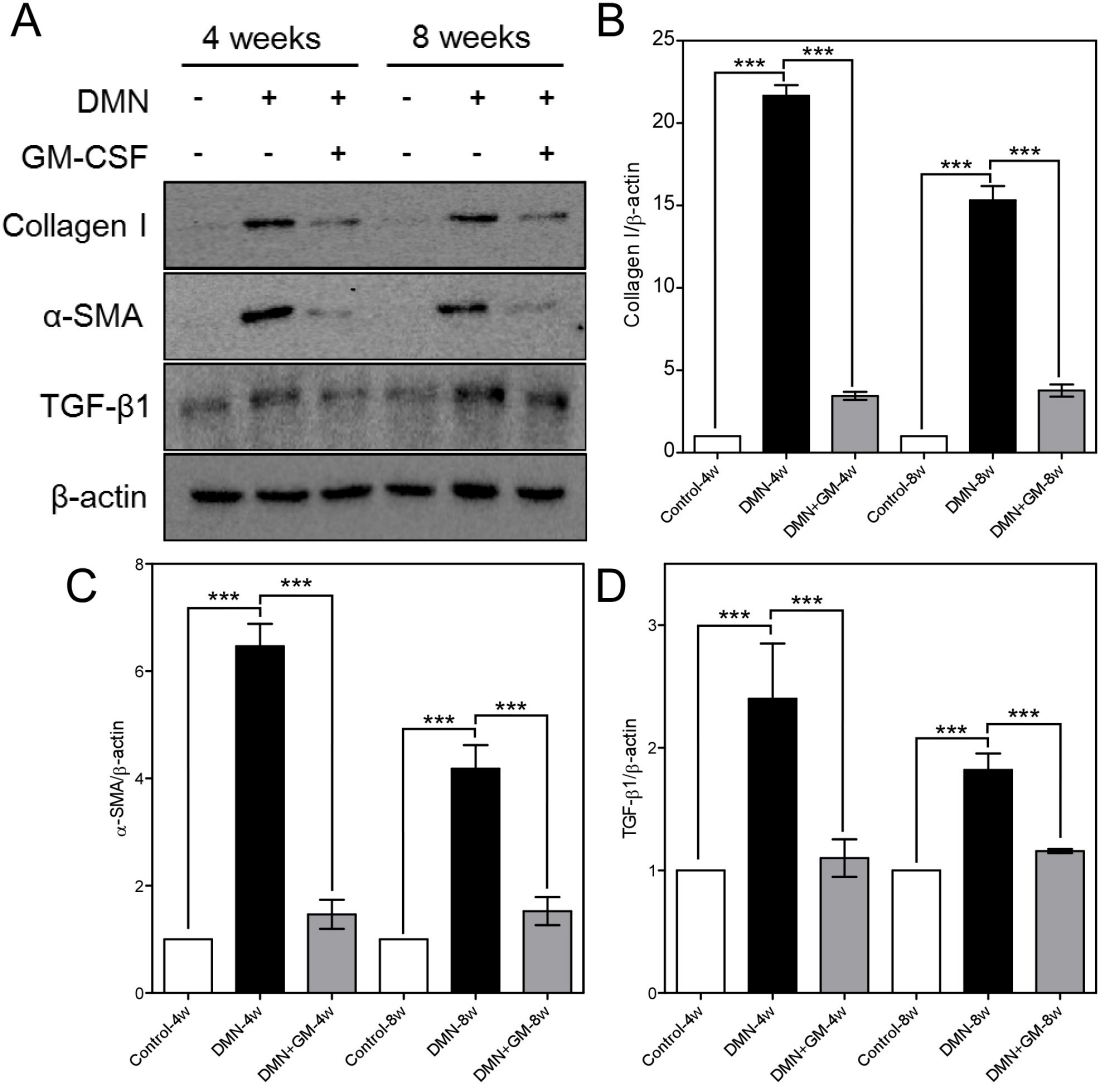
Western blot analysis of collagen type I, α-SMA, and TGF-β1 in liver. (**A**) Western blot images showing the expressions of collagen type I, α-SMA, and TGF-β1. β-Actin was used as the internal control. Gray density scanning results of (**B**) collagen type I, (**C**) α-SMA, and (**D**) TGF-β1 are shown. Results are expressed as means (± SEMs). Significant differences were observed between the control and DMN groups and between the DMN groups and the DMN+GM-CSF groups (***p ≤ 0.001).

Western blotting showed that DMN significantly increased collagen type I, α-SMA, and TGF-β1 levels and that GM-CSF significantly suppressed this increase in the DMN+GM-4w and -8w groups (Fig 9A). Fold increases versus corresponding controls of collagen type I (Fig 9B), α-SMA (Fig 9C), and TGF-β1 (Fig 9D) in the DMN+GM-4w and DMN-4w groups were 3.45 (± 0.25) and 21.66 (± 0.65), 1.47 (± 0.27) and 6.47 (± 0.41), and 1.1 (± 0.15) and 2.4 (± 0.45), respectively, and in the DMN+GM-4w and -8w groups were 3.78 (± 0.37) and 15.32 (± 0.87), 1.53 (± 0.26) and 4.18 (± 0.44), and 1.16 (± 0.02) and 1.82 (± 0.14) respectively.

## Discussion

DMN is a potent liver-specific toxin commonly used to induce liver fibrosis in animal models. Chronic administration of DMN to rats causes advanced liver fibrosis with diffuse nodularity, marked portal hypertension, and accumulation of ascites [32], and repeated injections induce the abnormalities and biochemical and pathological manifestations of liver injury leading to liver fibrosis. In the current study, DMN injections for 4 weeks induced liver fibrosis in rats, and this pathophysiology was maintained at 8 weeks without additional DMN injections. DMN-treated rat livers exhibited several characteristic pathological features of liver fibrosis, such as a shrunken, dark appearance with a hardened, rough surface and an abnormal arrangement of hepatic plates, infiltrating inflammatory cells, and collagen fiber deposition. In addition, serum biochemical indicators of liver inflammation such as AST, ALB, and TBIL were released into the bloodstream from the livers of DMN-treated rats.

The development and progression of liver fibrosis is a consequence of highly coordinated cellular and molecular processes following chronic liver injury that lead to the activation of HSCs. This activation from the quiescent state is considered a hallmark of hepatic fibrosis and results in collagen accumulation and ECM deposition in liver [33]. Furthermore, HSC activation is marked by highly upregulated levels of α-SMA [34], which leads to a myofibroblast-like phenotype and the deposition of large quantities of ECM components in liver [35]. Chronic exposure to DMN also increases α-SMA deposition, a widely accepted marker of the transactivation of HSCs to myofibroblasts [36]. In the present study, DMN injections increased the expressions of collagen type I and α-SMA protein in liver tissues in the DMN-4w and -8w groups, indicating HSCs activation.

TGF-β1 is a cytokine that directly activates HSCs and the fibrotic process [37]. Pathophysiologically, upon activation, TGF-β1 binds to its receptors and initiates the Smad-dependent and/or Smad-independent signaling pathways, which result in the upregulation of profibrotic genes, such as those encoding α-SMA and ECM proteins, and the secretions of cytokines and growth factors required for fibrosis [38]. DMN also activates TGF-β1 in rat liver [39]. We observed DMN markedly increased TGF-β1 expression in rat livers in the DMN-4w and -8w groups. Because of the significance of TGF-β1 activation in liver fibrosis progression, TGF-β1 pathway inhibition remains a therapeutic strategy for liver fibrosis [40].

GM-CSF has been shown to exert several pharmacological effects. In a previous study, we found GM-CSF inhibited TGF-β1-dependent collagen synthesis in a rabbit model of vocal fold scarring [28], and hypothesized that GM-CSF might also inhibit TGF-β1-dependent liver fibrosis. Consistent with this hypothesis, GM-CSF substantially reduced TGF-β1 expression in the livers of DMN-treated rats in the DMN-4w and -8w groups. Furthermore, immunohistochemical analyses showed TGF-β1 levels were significantly lower in DMN+GM groups than in DMN groups. In addition, we found that GM-CSF significantly prevented the DMN-induced decrease in PPARγ levels, which further confirmed the anti-hepatofibrotic effect of GM-CSF. It has been shown that the upregulation of PPAR-γ by GM-CSF is a prerequisite for the effect of GM-CSF because when PPAR-γ is activated/upregulated, it suppresses inflammation and inhibits the TGF-β1 signaling pathways. This inhibition of TGF-β1- increases α-SMA and collagen type I expression levels and ultimately reduces ECM deposition and ameliorates hepatic fibrosis in hepatic cells [41–43]. Based on these observations, we conclude that GM-CSF protects against DMN-induced rat liver fibrosis by inhibiting a TGF-β1 signaling pathway involving PPAR-γ. However, we acknowledge that we do not have direct evidence indicating which signaling pathway is responsible for the GM-CSF-induced inhibition of TGF-β1 expression in rat liver fibrosis. Nonetheless, we previously showed that GM-CSF blocks the Rho-ROCK signal downstream of TGF-β1 activation in a rat model of spinal cord injury [28]. Likewise, we have found that GM-CSF inhibited TGF-β1-induced collagen synthesis in human vocal fold fibroblast injury [20]. In this previous study, GM-CSF up-regulated hepatocyte growth factor (HGF) and its membrane-spanning tyrosine kinase receptor, c-Met, but did not regulate TGF-β1 expression. Notably, HGF expression favors the downregulation of collagen, whereas TGF-β1 increases the production of collagen type I and fibronectin. In another study, we showed GM-CSF activated major JAK-STAT, PI3K-AKT, and RAS-MAPK signal pathways [44, 45]. Thus, the molecular mechanism and the signaling pathway initiated by GM-CSF that leads to inhibition of the TGB-β1 pathway in liver fibrosis is complex. Additional studies are required to clarify the mechanisms involved.

The accumulation of ECM proteins has been reported to disturb liver architecture by forming fibrous scars and subsequently cirrhosis with nodules of regenerating hepatocytes, which often leads to progressive loss of liver function [3]. Hence, anti-fibrogenic therapies that suppress the activation of HSCs are considered an attractive means of preventing the pathological progression to cirrhosis in chronic liver diseases [46]. The results of the current study show that the number of α-SMA positive cells in liver was increased by DMN treatment, but that GM-CSF administration suppressed this increase and increased collagen accumulation. Taken together, these findings suggest that the antifibrotic effect of GM-CSF is due to the suppression of HSC activation. Moreover, GM-CSF inhibited DMN-induced liver damage and hepatotoxicity and increased body weights and survival rates. The hepatoprotective effects of GM-CSF were further confirmed by the prevention of DMN-induced liver weight loss. Furthermore, intraperitoneal GM-CSF injection maintained hepatic biomarkers and histological integrity at near normal levels.

Some rats died in the DMN+GM-8w group, in which animals were injected with DMN for the first 4 weeks and GM-CSF for the following 4 weeks, and most of the rats that died succumbed before the end of the 4^th^ week, and thus were not treated with GM-CSF. On the other hand, in the DMN+GM-4w group, in which GM-CSF and DMN were co-administered, GM-CSF considerably minimized the toxic effects of DMN. It is not clear why GM-CSF post-treatment improved histology and gene expression levels but not survival rate. We speculate that the therapeutic effects of GM-CSF at the molecular and cellular levels were insufficient to improve survival rate in the sequential treatment model. Therefore, it would appear that the therapeutic effects of GM-CSF, including its effect on survival rate, depend on the pathophysiology of liver injury and that GM-CSF should be administered as soon as possible after liver injury.

Several earlier studies have also reported that GM-CSF has hepatoprotective effects [25, 47]. Importantly, exogenous GM-CSF administration has been reported to increase proliferative indices in normal livers and to promote hepatocellular DNA synthesis [25]. In addition, GM-CSF-deficient mice were found to develop hepatosteatosis resembling nonalcoholic fatty liver disease [48]. However, other studies have reported hepatoprotective effects for anti-GM-CSF agents [49, 50], and thus, reported results in the literature conflict regarding the effects of GM-CSF on liver fibrosis. However, it should be noted that the pathophysiology of liver fibrosis is a dynamic and progressive process involving diverse, complicated mechanisms and that these studies were performed using various liver disease models in animals and humans. The inflammatory state of liver fibrosis changes significantly during the progression of liver fibrosis and the subsequent formation of hepatocellular carcinoma (HCC) [51]. The temporal inflammatory status of fibrotic liver tissues and livers with HCC tumors is an important factor that dictates therapeutic strategies. For example, anti-angiogenic therapy for liver fibrosis works only during the early-stage liver fibrosis and does not reverse late-stage disease [52]. Hence, the choice of GM-CSF or anti-GM-CSF therapy depends on liver fibrosis status and other factors, such as the type of disease model used.

## Conclusion

In summary, our results confirm the inhibitory and therapeutic effects of GM-CSF against DMN-induced liver fibrosis in rats. Our results show that GM-CSF had specific therapeutic effects on pathological changes in liver. Furthermore, the study demonstrates that GM-CSF acts as anti-fibrogenic agent and can significantly reduce the DMN-induced increases in collagen type I and α-SMA, possibly by suppressing TGF-β1 expression and increasing PPAR-γ expression, which might ultimately suppress HSC activation. However, we did not address the molecular mechanism responsible for the effects of GM-CSF, though we speculate it might depend on one or more diverse cellular actions of GM-CSF, such as stem cell activation, enhanced immune cell functionality, or direct inhibition of liver fibrosis. In practice, it is difficult to address this subject due to the complicated pathophysiology of liver fibrosis. Nevertheless, further studies will undoubtedly improve understanding and provide important clues.
